# Combination Therapy With Beta‐Blockers and Angiotensin‐Converting Enzyme Inhibitors for Cardiovascular Diseases: Focus on Bisoprolol/Ramipril

**DOI:** 10.1155/cdr/6773898

**Published:** 2026-05-27

**Authors:** Agostino Virdis

**Affiliations:** ^1^ Department of Clinical and Experimental Medicine, University of Pisa, Pisa, Italy, unipi.it

**Keywords:** angiotensin-converting enzyme inhibitors, atrial fibrillation, beta-blockers, coronary artery disease, heart failure, hypertension

## Abstract

Patients with hypertension, especially when complicated by cardiovascular disease (CVD) and/or comorbidities, require long‐term treatment with a combination of antihypertensive drugs, which often gives rise to complex drug regimens. Dual‐agent combination antihypertensive therapy is recommended in most patients with hypertension, and where available, a fixed‐dose single‐pill combination (SPC) is recommended. The combination of antihypertensive drugs should take into account the patient′s overall CVD risk profile (other CVD risk factors and diagnoses). The combination of a beta‐blocker and angiotensin‐converting enzyme inhibitor (ACEi) acts on two neuroendocrine systems implicated in the pathophysiology of hypertension and multiple CVD: beta‐blockers on the sympathetic nervous system and ACEis on the renin–angiotensin system (RAS). A large body of solid evidence states that, in addition to their antihypertensive effects, both beta‐blockers and ACEis provide cardioprotection and guarantee reductions in CV morbidity and mortality in patients with coronary artery disease (CAD), atrial fibrillation, and heart failure. For these reasons, such a combination might be beneficial for many patients. This review summarizes the evidence supporting the use of beta‐blockers and ACEis, as well as the rationale behind their combined administration, particularly in patients with a high risk of CVD, such as those with ischemic heart disease, atrial fibrillation, or heart failure.

## 1. Introduction

Cardiovascular disease (CVD) accounts for approximately one‐third of all deaths globally [1]. Antihypertensive agents are a well‐established pharmacotherapy across the CVD spectrum. They are used in the management of patients with hypertension, a main CVD risk factor [1], and patients with CVDs such as coronary artery disease (CAD) and cardiac arrhythmia (e.g., atrial fibrillation [AF] and heart failure [HF]).

There are five major classes of antihypertensive agents: angiotensin‐converting enzyme inhibitors (ACEis), angiotensin receptor blockers (ARBs), dihydropyridine calcium channel blockers (CCBs), thiazide and thiazide‐like diuretics, and *β*‐adrenoceptor blockers (beta‐blockers) [2]. Different antihypertensive classes are associated with different cardiovascular outcome benefits, and they are therefore frequently used in various combinations [3, 4]. As drug regimens grow in complexity, particularly for patients with multiple cardiovascular risk factors and CVD diagnoses, poor treatment adherence becomes a significant concern [5].

Reducing the drug regimen burden through the use of single‐pill combination (SPC) therapy has well‐described advantages, including combining different drug classes to leverage different mechanisms of action, leading to improved patient treatment adherence and cardiovascular prognosis [5]. Many different SPCs of dual antihypertensive combinations are currently available in Europe. In 2016, the first SPC of a beta‐blocker and ACEi (bisoprolol/perindopril) was approved for use in patients with hypertension, stable CAD, and/or HF [6]. Recently, a fixed‐dose SPC of bisoprolol (beta‐blocker) with ramipril (ACEi) was developed [7]. This bisoprolol/ramipril SPC is currently approved in several European countries (Poland, Czechia, Slovakia, and Italy) for use in various cardiology indications [8].

This review describes the role of beta‐blocker/ACEi combination therapy, with a focus on bisoprolol/ramipril, in four important conditions in the CVD continuum: hypertension, CAD, AF, and HF. Where data describing combination treatments are lacking, evidence for the individual agents or classes is discussed. This review takes a narrative approach and is not intended to be an exhaustive review of all the literature to date.

## 2. Data Sources

To identify the data reviewed herein, a search of PubMed was undertaken using the terms “beta‐blocker∗” and “ACE inhibitor∗” (and synonyms) [Text Word] in association with “combination” (and synonyms) [Title]. An additional search using these terms was limited to meta‐analyses, in order to specifically capture any meta‐analyses of this combination. All English‐language articles were identified up to June 6, 2025, with no initial date limit set. Relevant literature was also identified based on ad hoc searches for specific indications, as well as by reviewing the reference lists of identified articles.

## 3. Hypertension

Hypertension is a key risk factor for CVD and remains highly prevalent, with an age‐standardized prevalence of at least 20% in both sexes across countries of Central and Eastern Europe, Central and South Asia, and sub‐Saharan Africa [1]. The risk of CVD or CVD‐related death increases as systolic blood pressure (SBP) and diastolic blood pressure (DBP) increase [9, 10]. A study in over 1.25 million individuals found a strong positive association between elevated SBP or DBP and various CVDs, with a significantly increased lifetime risk of overall CVD among individuals with hypertension versus without (63.3% vs. 46.1%) [10]. Risk of CVD death increased as blood pressure (BP) increased for any SBP > 115 mmHg and any DBP > 75 mmHg in a large meta‐analysis (61 studies) [9].

Deaths due to CVD are largely preventable through the effective treatment of hypertension [11]. For every 10‐mmHg reduction in SBP, CVD risk declines overall by 20%, although there is some variance in this risk reduction by type of CVD (stroke vs. hypertensive HF vs. ischemic heart disease [IHD]) [12]. The benefits of lowering BP are well established [13–16].

Despite this evidence, hypertension treatment rates globally remain disappointing (47% in women, 38% in men) [17]. Of additional concern is the relatively high rate of untreated hypertension (50%) among young adults [18]. Furthermore, antihypertensive treatment adherence is another common, significant concern in the management of patients with hypertension [2, 19], since poor adherence is associated with an increased risk of cardiovascular complications [20].

Among the many options to improve hypertension treatment rates and adherence to treatment, use of SPCs is advocated [2, 19], since most patients will require a combination of at least two antihypertensive agents to achieve BP control [21].

### 3.1. Combination Therapy as Initial Therapy

The overall rationale for combining antihypertensives is that there is a synergistic effect of the hypertensive action if different drug classes are combined and, if the drugs are used at lower doses than when given as monotherapy, a potential overall reduction in adverse effects. Indeed, the benefit to BP reduction of dual combination therapy exceeds that of increasing a monotherapy dose, with a fivefold greater reduction in BP observed with combination therapy [14]. Both the 2024 European Society of Cardiology (ESC) and the 2023 European Society of Hypertension (ESH) guidelines for hypertension management recommend initial dual‐agent combination therapy in most patients with hypertension, with a number of exceptions (see below) [2, 19]. Importantly, where available, a fixed‐dose SPC is preferred [2, 19]. Exceptions to initial dual‐agent combination therapy include frail patients, very old patients, patients with modestly elevated BP (exception in both guidelines [2, 19]), very high‐risk patients with high‐normal BP (an exception in the ESH guidelines [19]), or patients with symptomatic orthostatic hypotension (an exception in the ESC guidelines [2]). In contrast, American guidelines recommend initial therapy with a combination of two different classes (either as separate agents or as a fixed‐dose combination) only in adults with Stage 2 hypertension (BP ≥ 140/90 mmHg) who have an average BP reading of > 20/10 mmHg above their BP target [22]. If initial monotherapy is not effective in achieving the target BP, additional agents can be added as required.

Results from a recent Monte Carlo simulation study using data from 1.1 million individuals with hypertension eligible for dual antihypertensive combination therapy confirmed that the risk of long‐term cardiovascular outcomes is successfully reduced by sustained guideline‐directed dual antihypertensive therapy [23]. In principle, provided two different drug classes are combined, any combination of the five major antihypertensive drug classes can be used as initial combination therapy [2, 19]. Exceptions are (i) the combination of the two renin–angiotensin system (RAS) blocker classes (ACEi + ARB) and (ii) the combination of a direct renin inhibitor plus an ACEi or ARB.

Selecting the appropriate combination of antihypertensive agents involves consideration of the patient′s overall CVD risk profile, including the presence of other CVD risk factors and/or comorbidities. In Europe, the preferred class combinations for treating hypertension are a RAS inhibitor (ACEi or ARB) plus either a CCB or a thiazide/thiazide‐like diuretic [2, 19]. Importantly, both the ESC and ESH hypertension guidelines identify specific subgroups of patients who should receive dual therapy with a beta‐blocker plus another agent, and these groups are broadly similar in both guidelines [2, 19]. They include patients with concomitant angina, patients post–myocardial infarction (MI), patients with HF with reduced ejection fraction (HFrEF), and patients who require heart rate control (e.g., AF) [2, 19]. Additional subgroups identified by the ESH for beta‐blocker dual therapy include patients with acute or chronic coronary syndromes (ACS or CCS), women of child‐bearing potential and/or who are planning a pregnancy, or patients with a hypertension disorder in pregnancy [19, 24].

**Figure 1:**
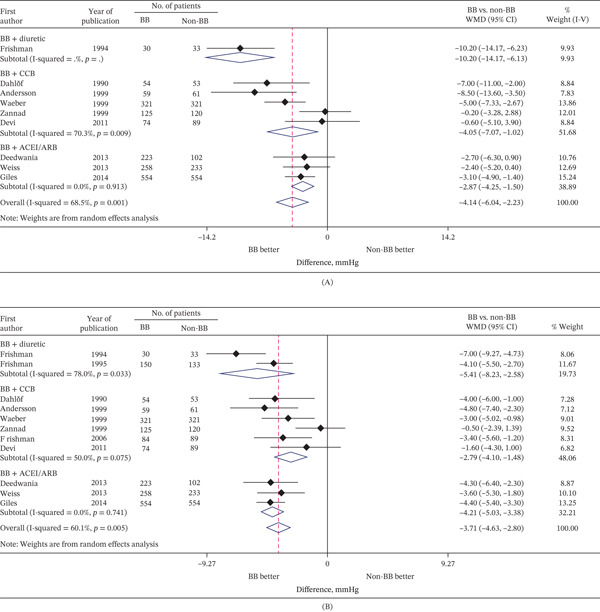
Treatment effects of beta*-*blocker add‐on therapy on (A) systolic and (B) diastolic blood pressure in the sitting or supine position. Adapted from Figure 2 in Guo et al. [25], which was published under a Creative Commons CC‐BY 4.0 License (adaptation was removal of part (C) from the original figure). See the original publication for full reference citations for the studies included in these figures. Black symbols represent the point estimate of each trial. Horizontal lines denote 95% CIs of each trial. Diamonds represent the overall or subtotal pooled estimate and 95% CI of trials. Abbreviations: ACEi, angiotensin‐converting enzyme inhibitor; ARB, angiotensin receptor blocker; BB, beta‐blocker; CCB, calcium channel blocker; CI, confidence interval; WMD, weighted mean difference.

The BP‐lowering effect of adding a beta‐blocker (excluding atenolol) to monotherapy with a different class of antihypertensive agent was significantly greater than the non–beta‐blocker monotherapy, according to a meta‐analysis of randomized controlled trials (RCTs) in patients with hypertension [25]. For example, the weighted mean difference in SBP was −2.9 mmHg (95% confidence interval [CI] −4.3, −1.5) and in DBP was −4.2 mmHg (95% CI −5.0, −3.4) between a beta‐blocker plus ACEi/ARB versus ACEi/ARB monotherapy (Figure 1). Achieving the SBP target was more likely in the add‐on beta‐blocker population than the non–beta‐blocker monotherapy population (odds ratio [OR] 1.34, 95% CI 1.12–1.61) [25].

### 3.2. Beta‐Blocker + ACEi Combination

There is a strong pharmacodynamic rationale for the combination of a beta‐blocker with an ACEi in patients with hypertension [6]. Beta‐blockers target the sympathetic nervous system (SNS) to reduce cardiac output, and ACEis act on the RAS to promote vasodilation and reduce vascular resistance [6]. These two neuroendocrine systems are implicated in the pathophysiology not only of hypertension but also of CVDs along the CVD continuum [6]. To the best of my knowledge, RCTs in patients with hypertension comparing a beta‐blocker/ACEi combination to other drug combinations, either from the beginning of the trial or as a later addition to initial monotherapy, have not been conducted.

Observational data are, however, available for this drug class combination. Clinical studies assessing bisoprolol in combination with ramipril were not identified in the literature search; however, data are available from bisoprolol plus perindopril studies (Table 1) [26–30]. The bisoprolol/perindopril fixed‐dose combination or SPC was investigated in four studies [26–29], and the free combination in a post hoc analysis of data pooled from three observational trials [30]. As expected (see Table 1), the combination of a beta‐blocker plus ACEi effectively reduced BP and improved heart rate in patients who also had CAD [26], a history of revascularization for ACS [28] or CAD [27, 29]. Reductions in angina attacks and nitrate consumption were also seen in patients with stable CAD [26, 27, 29]. Supporting data from a retrospective analysis of real‐world clinical practice data based on the Italian National Health Service database found that ramipril and bisoprolol were the most commonly dispensed individual compounds within the ACEi and beta‐blocker classes, respectively, between 2013 and 2019 [34]. Data analysis allowed the authors to conclude that the population who made continuous use of the ramipril–bisoprolol combination was predominantly male and elderly, with the trend in dispensing showing the highest value among the very elderly. The authors emphasized the importance of making ramipril and bisoprolol available as a fixed‐dose SPC to simplify treatment and improve adherence, especially among the elderly, which would be expected to reduce hospitalizations for preventable events and subsequently lower costs for the National Health Service [34]. Another advantage of using a beta‐blocker/ACEi fixed‐dose combination or SPC is the certainty of adequate drug dosages for reducing abnormal RAS and SNS activation in patients with hypertension and left ventricular dysfunction [35].

**Table 1:** Studies in patients with hypertension (±CVD) receiving a combination of a beta‐blocker and ACEi.

Study acronym, author (year)	Study design (duration)	Patient population	Bisoprolol/perindopril (mg/day)	BP outcomes	HR outcomes	Clinical outcomes	Safety
Single‐pill combination							
PRIDE, Kobalava et al. (2023) [26]	Ambispective, OB, multicenter (12 weeks)	Arterial HT, stable CAD, history of MI (*n* = 481) Mean ± SD age: 61.4 ± 8.9 years	SPC 2.5/2.5, 5.0/5.0, 10.0/10.0 (±CCB, diuretic, trimetazidine, ivabradine, nicorandil)	Reduction in SBP/DBP (mean): −24.9/−12.2 (*p* < 0.001 vs. b/l). Target BP (% pts): 95.9%	Target HR (% pts): 34.5% (vs. 3.1% at b/l, *p* < 0.001)	Reductions in: ‐ % pts with angina ‐ No. of angina attacks ‐ Nitrate consumption	No AEs or withdrawals due to AEs
STYLE, Boytsov et al. (2021) [27]	Prospective, OB, multicenter (12 weeks)	Arterial HT, stable CAD (*n* = 1892) Mean ± SD age: 61.9 ± 8.8 years	SPC 2.5/2.5, 5.0/5.0, 5.0/10.0, 10.0/10.0 (±CCBs, beta‐blockers, ACEis, ARBs, imidazole receptor agonists)	Reduction in SBP (mean ± SD): −31.5 ± 14.2 mmHg (*p* < 0.0001 vs. b/l) Reduction in DBP (mean ± SD): −15.9 ± 9.5 mmHg (*p* < 0.0001 vs. b/l) Target BP (% pts): 86.7%	Target HR (% pts): 24.7% (vs. 1.5% at b/l)	Reductions in: ‐ No. of angina attacks ‐ Nitrate consumption ‐ Resting HR	Nine AEs in 4 pts, resulting in withdrawal of study drug in 3 pts
Korennova et al. (2019)^e^ [28]	Prospective, OB, single center (12 weeks)	Arterial HT, postrevascularization for ACS on standard therapy and target BP not achieved on ACEi + beta‐blocker free combination (*n* = 91)^a^ Mean ± SD age, years: 61.5 ± 9.7	SPC 5/5, 5/10, 10/10	Target SBP/DBP (% pts): 92.0% at Week 4 and 100.0% at Week 12	Target HR (% pts): 95.4% at Week 4 and 100% at Week 12	Reductions in: ‐ No. of angina attacks (among pts with CHF and/or angina, *n* = 41)	No AEs reported
Fixed‐dose combination							
PRESTOL, Lutai and Golikova (2019)^e^ [29]	OB, multicenter (4 weeks)	Arterial hypertension, CAD on bisoprolol 1.25–10 mg monotherapy (*n* = 2394)^b^ Mean age: 61.4 years	FDC^c^ 5/10, 5/5, 10/10 (±statins, antiplatelet agents, CCBs, diuretics, nitrates)	Baseline SBP/DBP (mean) 159.3/94.6 mmHg Week 4 SBP/DBP (mean) 131.3/80.5 mmHg Target SBP/DBP (% pts): 86.9%	Baseline HR (mean ± SD) 81.6 ± 10.1 bpm Week 4 HR (mean) 64.8 bpm Target HR (% pts): ≤ 70 bpm, 84.9%; ≤ 60 bpm, 31.6%	Reductions in: ‐ No. of angina attacks per week ‐ % pts reporting angina pain ‐ Weekly nitroglycerin use	AEs in 9/2250 pts (0.4%): Hypotension 0.2%; cough 0.2%; bradycardia 0.08%; diarrhea 0.08%
Free combination							
CONFIDENCE II, PROTECT I, PROTECT III, Abeel et al. (2022) [30]	Post hoc analysis of three OB trials^d^ (16 weeks)	Mild‐to‐moderate HT (140 ≤ SBP ≤ 179 mmHg and/or 90 ≤ DBP ≤ 109 mmHg or 130 ≤ SBP ≤ 179 mmHg and/or 80 ≤ DBP ≤ 109 mmHg + DM, renal disease, or proteinuria) (*n* = 845) Mean ± SD age: 68.3 ± 11.3 years	Bisoprolol 2.5, 5, or 10 mg/day + perindopril 4 or 8 mg/day (±CCB, diuretics)	Reduction in SBP (mean ± SD) −14.7 ± 12.3 mmHg (*p* < 0.001 vs. b/l) Reduction in DBP (mean ± SD) −6.7 ± 8.2 mmHg (*p* = 0.059 vs. b/l) Target BP (% pts): 78.0%	No statistically significant change in HR	—	AEs: 7.1% of pts Discontinuation due to AEs: 1.1% of pts

Abbreviations: ACEi, angiotensin‐converting enzyme inhibitor; ACS, acute coronary syndrome; AE, adverse event; ARB, angiotensin receptor blocker; b/l, baseline; BP, blood pressure; CAD, coronary artery disease; CCB, calcium channel blocker; CHF, chronic heart failure; CVD, cardiovascular disease; DBP, diastolic blood pressure; DM, diabetes mellitus; FDC, fixed‐dose combination; HR, heart rate; HT, hypertension; LVEF, left ventricular ejection fraction; MI, myocardial infarction; No., number; NYHA, New York Heart Association; OB, observational; pts, patients; RAAS, renin–angiotensin–aldosterone system; SBP, systolic blood pressure; SD, standard deviation; SPC, single‐pill combination.

^a^Standard therapy was dual antithrombotic treatment, statin(s), RAAS inhibitors, and beta‐blockers, with or without anticoagulant(s), CCBs, and diuretics, with or without physical rehabilitation therapy [28]. Additional inclusion criteria were as follows: absence of contraindication to participate in physical rehabilitation and failure to achieve adequate BP and HR response to exercise, in combination with Class II–III angina and/or CHF with preserved LVEF, NYHA Class II–III while on well‐tolerated free combinations of ACEis and beta‐blockers [28].

^b^The number of patients enrolled in this study was not directly specified [29]; 170 outpatient cardiologists enrolled 15 patients each (corresponding to 2550 patients according to this author′s calculation). At the baseline study visit, participating cardiologists completed a case report form for each patient and assessed their treatment adherence using a questionnaire, followed by clinical assessment and AE recording at the Week 4 visit (study end). Of the 2785 patient questionnaires submitted, 2394 met study inclusion criteria [29]; it has therefore been inferred that 2394 patients were included in PRESTOL.

^c^It was not clear from the manuscript whether an SPC was used, although the Introduction to the study mentioned the recent marketing approval for an FDC SPC of bisoprolol/perindopril in Ukraine [29]. Three dose levels are described, but other dose levels (undescribed) were used in a minority (2.4%) of patients [29].

^d^The three studies included in this post hoc analysis were prospective, open‐label, multicenter observational studies: CONFIDENCE II [31], PROTECT I (data available only as an abstract) [32], and PROTECT III (data available only as an abstract) [33].

^e^Lutai and Golikova (2019) translated from Ukrainian and Korennova et al. (2019) from Russian.

Evidence for the antihypertensive efficacy of bisoprolol was established in pivotal trials in patients with mild‐to‐moderate hypertension; bisoprolol 5–20 mg/day was as effective as atenolol 50–100 mg/day, low‐dose metoprolol (100 mg/day), and the CCB nifedipine 40–80 mg/day, and more effective than diuretics (hydrochlorothiazide + amiloride) (as reviewed previously [36]). Similarly, ramipril is a well‐known ACEi, with its antihypertensive efficacy established in early trials of patients with mild‐to‐moderate hypertension. Ramipril had similar antihypertensive efficacy to other ACEis (enalapril, captopril, and lisinopril) and the beta‐blocker atenolol (see review by Frampton and Peters [37]).

## 4. CAD

CAD refers to a range of related chronic and acute conditions. CCS (also referred to as coronary heart disease [CHD]) is comprised of stable chronic angina, obstructive/nonobstructive CAD with or without prior MI or revascularization, and IHD [38]. ACS includes acute MI (AMI) or unstable angina, where AMI is further divided between non–ST‐elevation MI (NSTEMI) and ST‐elevation MI (STEMI) [39]. It was recently proposed that a new binary classification be adopted, using the terms “acute myocardial ischemic syndromes” and “nonacute myocardial ischemic syndromes” to align terminology with the different pathophysiological causes of myocardial ischemia; however, this has yet to be adopted in major clinical guidelines from the ESC, ACC, or AHA [40].

CAD is the most prevalent of CVDs globally, with over 135 million cases according to the 2022 Global Burden of Disease study data [41]. CAD is associated with the highest level of morbidity compared with other CVDs, with an age‐standardized rate of disability‐adjusted life years (DALYs) of 2275.9 per 100,000 [41]. As such, CCS treatment goals are to reduce ischemia and angina symptoms, prevent cardiovascular events and death, and improve patient quality of life [38, 42], in which antihypertensive therapy plays an important role.

Antihypertensive therapy may be required after an ACS event for the long‐term management of the patient and for secondary prevention [39, 43]. Since hypertension commonly co‐occurs with CCS and individuals with both are considered to be at very high risk of future atherosclerotic‐related CVD events [38], antihypertensive therapy is recommended to reduce the risk of cardiovascular events/mortality [38, 42]. Beta‐blockers or RAS inhibitors (ACEis or ARBs) are included among initial treatment options for patients with CCS and hypertension, particularly those with a recent history of MI [38, 42]. In European guidelines, patients with symptomatic angina can be prescribed a beta‐blocker and/or a CCB [42]. As with any antihypertensive treatment strategy, additional agents can be added if target BP levels are not achieved or angina symptoms are not adequately controlled.

Similarly, long‐term management of cardiovascular risk after an ACS event includes the use of ACEis and beta‐blockers, regardless of hypertension status [39]. Beta‐blockers are specifically recommended for patients with reduced left ventricular ejection fraction (LVEF), and ACEis specifically for patients with diabetes, chronic kidney disease, hypertension, or clinical HF and/or reduced LVEF (≤ 40%) [39].

### 4.1. Beta‐Blocker + ACEi Combination

Evidence of the efficacy of a beta‐blocker with an ACEi was demonstrated in patients with stable CAD (in a subgroup analysis of the EUROPA trial; *n* = 7534) [44] and in patients after an AMI (in observational registry studies) [45, 46]. In the EUROPA trial, perindopril plus beta‐blocker therapy significantly reduced the risk of composite cardiovascular events by 24% (hazard ratio [HR] 0.76; 95% CI 0.64–0.91; *p* = 0.002), hospitalization for HF (HHF) by 45% (HR 0.55; 95% CI 0.33–0.93; *p* = 0.025), and fatal or nonfatal MI by 28% (HR 0.72; 95% CI 0.59–0.88; *p* = 0.001) versus patients receiving a beta‐blocker alone [44]. Overall and cardiovascular‐related mortality rates were not significantly different between groups. In the Spanish registry study (PRIAMHO‐II, *n* = 5397), patients receiving combination therapy at the time of discharge after an AMI had a significantly reduced risk of 1‐year mortality (HR 0.51; 95% CI 0.32–0.82; *p* < 0.05); this benefit was not observed with either agent alone [45]. When patients were grouped by cardiovascular risk status, this benefit with the combination was observed only in patients with high CV risk (LVEF < 40% or Killip Class 1) [45].

**Figure 2:**
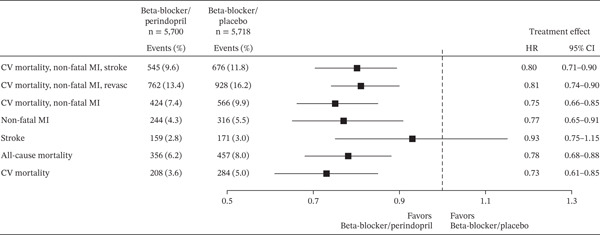
Treatment effect of the combination of beta‐blocker plus ACEi versus beta‐blocker plus placebo: a forest plot. Reproduced from Figure 4 in Brugts et al. [47], which was published under a Creative Commons CC‐BY 4.0 License. A Cox regression multivariate analysis was performed to calculate HRs and 95% CIs with adjustments for the full model. The primary endpoint was the composite endpoint of cardiovascular mortality, nonfatal MI, and stroke. Among the 11,418 patients taking a beta‐blocker, 5700 were randomized to a perindopril‐based regimen and 5718 to placebo. The *p*‐interaction was significant for all‐cause mortality and CV mortality; all other *p*‐interactions are not significant. Abbreviations: ACEi, angiotensin‐converting enzyme inhibitor; CI, confidence interval; CV, cardiovascular; HR, hazard ratio; MI, myocardial infarction.

While serving as less direct specific evidence, a combined analysis of individual data from the ADVANCE, EUROPA, and PROGRESS trials confirmed that a beta‐blocker plus ACEi combination provides cardioprotective effects (except for stroke risk reduction) in patients with vascular disease (Figure 2) [47].

### 4.2. Beta‐Blockers

Pivotal evidence on the use of beta‐blockers in patients with MI (acute or past) found an overall reduced risk of death over the long term (~2 years) in two meta‐analyses [48, 49]. However, in one of these meta‐analyses, only a minority of patients had reduced LVEF (and/or HF), limiting the conclusions that could be drawn regarding the effect of beta‐blockers post‐MI in patients with reduced LVEF (< 40%) [49]. Reduction in mortality risk conferred by beta‐blocker treatment for patients with AMI may depend on the specific beta‐blocker used, as suggested in results from a meta‐analysis of carvedilol versus *β*_1_‐selective beta‐blockers (bisoprolol, atenolol, metoprolol, and nebivolol) [50]. Carvedilol was superior to the *β*_1_‐selective beta‐blockers for all‐cause mortality risk reduction but not for nonfatal MIs or HF readmissions in patients with AMI [50].

A recent open‐label study (REDUCE‐AMI) confirmed that there is little benefit of initiating long‐term beta‐blocker treatment postcoronary angiography for AMI in 5020 patients with preserved LVEF (≥ 50%) [51]. After a median follow‐up of 3.5 years, there was no significant difference in the incidence of the primary endpoint of a composite of all‐cause death or new MI (HR 0.96; 95% CI 0.79–1.16; *p* = 0.64) [51].

As for patients with stable CAD, while guidelines recommend beta‐blocker treatment for selected patient populations (e.g., reduced LVEF or with symptoms of angina; see above), a recent Canadian retrospective cohort study demonstrated significant clinical benefit in a broad IHD population [52]. Patients with stable IHD initiating beta‐blocker treatment (*n* = 12,695) were included and compared with patients with stable IHD not receiving beta‐blocker therapy (*n* = 15,344; control group). Overall, the most commonly used beta‐blocker was bisoprolol (66% of patients). There was a significantly lower 5‐year risk of the primary cardiovascular outcome (a composite of all‐cause death, HF hospitalization, or MI) with beta‐blocker treatment compared with controls (HR 0.92; 95% CI 0.86–0.98; *p* = 0.006), with a respective incidence of the primary outcome of 14.3% versus 16.1% [52].

As for reducing cardiovascular risk in patients with angina, results of a recent large database cohort analysis (*n* = 7607) with long‐term follow‐up (up to 14 years) were consistent with guideline recommendations for the use of beta‐blockers [53]. Bisoprolol was superior to other beta‐blockers (HR 0.45; 95% CI 0.34–0.61) and other pharmacological agents (HR 0.50; 95% CI 0.38–0.66) for the reduction of mortality risk, as well as cardiovascular events (angina and MI) [53].

### 4.3. ACEis

The cardioprotective benefit from ACEi treatment extends across the spectrum of patients with CCS, according to several important trials. This includes patients with stable vascular disease, CAD, or HF as demonstrated in the PEACE [54] and EUROPA [55] trials and high‐risk patients with vascular disease (i.e., CAD/stroke/peripheral vascular disease/diabetes plus one other cardiovascular risk factor such as hypertension) as shown in the Heart Outcomes Prevention Evaluation (HOPE) trial (of the ACEi ramipril) [56, 57]. Data from these three trials were pooled for meta‐analysis [58], which confirmed that ACEis significantly reduced all‐cause and cardiovascular‐related mortality versus placebo (7.8% vs. 8.9% [*p* = 0.0004] and 4.3% vs. 5.2% [*p* = 0.0002], respectively). The incidence of most cardiovascular events was also significantly reduced in ACEi recipients versus placebo recipients: nonfatal MI (5.3% vs. 6.4%; *p* = 0.0001), stroke (2.2% vs. 2.8%; *p* = 0.0004), HF (2.1% vs. 2.7%; *p* = 0.0007), and coronary–artery bypass surgery (6.0% vs. 6.9%; *p* = 0.0036) [58].

## 5. AF

AF is a prominent public health burden; around 52 million individuals globally have AF or atrial flutter (AFL), resulting in 8.36 million DALYs (2021 data) [59]. One of the key treatment goals is to control heart rate, with the target rate dependent on the treatment setting (acute/long term), symptom burden, and presence of HF [60]. The overall approach to patient management must also take into account cardiovascular risk factors and comorbidities. To achieve heart rate control in the acute setting, the ESC recommends treatment with beta‐blockers (regardless of LVEF status) or diltiazem/verapamil (for patients with an LVEF > 40%) [60]. Selective *β*_1_‐adrenoceptor blockers, such as bisoprolol, are preferred over unselective beta‐blockers because of their superior efficacy and safety in acute AF [60, 61]; selective *β*_1_‐adrenoceptor blockers offer many advantages in this setting. Given that *β*_1_ receptors are prevalent in the sinoatrial and atrioventricular nodes, selective *β*_1_‐adrenoceptor blockers are particularly effective at slowing conduction and reducing the ventricular rate in patients with AF [62]. In addition, *β*_1_ selectivity has advantages when other conditions are present: For instance, as selective *β*_1_‐adrenoceptor blockers have less of an effect on bronchial *β*_2_ receptors, they reduce the risk of bronchospasm in patients with chronic obstructive pulmonary disease or asthma [63–65]. Furthermore, *β*_1_ selectivity results in less peripheral vasoconstriction in patients with peripheral vascular disease [66]. Long‐term heart rate control is achieved using beta‐blockers, CCBs, digoxin, or a combination of these [60].

Beta‐blockers are effective antiarrhythmia agents, not only in AF but also in other arrhythmias that commonly occur in HF and MI [67]. Arrhythmias are associated with cardiac remodeling including cardiomyocyte hypertrophy and increased fibrosis, which are also features of CVD [67]. Beta‐blockers act by blocking *β*‐adrenergic receptor stimulation on cardiomyocytes in the heart muscle induced by adrenaline (from the adrenal medulla) and noradrenaline (from cardiac sympathetic nerves) [67]. By slowing conduction through the atrioventricular node, they provide ventricular rate control in AF. Bisoprolol is effective for heart rate control during AF (as reviewed in depth previously) [68]. It is unclear whether effective heart rate control has mortality benefits.

In patients with chronic HF in the Cardiac Insufficiency Bisoprolol Study (CIBIS) II trial, bisoprolol did not significantly reduce mortality risk versus placebo in patients with concomitant AF (relative risk 1.16; *p* = not significant) [69, 70]. Nevertheless, in the BISO‐CAD Phase IV study, patients with CAD (*n* = 866) treated with bisoprolol experienced a reduction in resting heart rate, and composite cardiovascular events (cardiovascular death, nonfatal AMI, hospitalization for unstable angina, or revascularization) occurred more commonly in patients with a heart rate of 69–72 versus < 65 bpm [71]. However, there was no significant correlation between heart rate and composite cardiovascular events in the intention‐to‐treat population, only in the efficacy analysis subgroup (*p* = 0.0412) [71].

Some evidence shows that inhibition of the RAS as add‐on therapy can help prevent AF recurrence [72]. RAS inhibition may reduce the structural and electrical cardiac remodeling associated with AF and as such might be considered to have antiarrhythmic properties [73]. Therefore, it would seem rational to combine an ACEi with a beta‐blocker when a patient with AF has concomitant hypertension. Indeed, antihypertensive therapy is recommended to lower BP as a component of the overall strategy to treat AF, reduce AF recurrence/progression, and prevent cardiovascular events [60]. A meta‐analysis found a reduced risk of new‐onset AF (OR 0.81; 95% CI 0.67–1.00; *p* = 0.037) or recurrent AF (OR 0.46; 95% CI 0.33–0.62; *p* ~0.001) associated with RAS inhibition in patients with hypertension [74]. Antihypertensive therapy with an RAS inhibitor in elderly patients (mean age 80.8 years) with AF on oral anticoagulants reduced mortality risk (HR 0.758; 95% CI 0.612–0.940; *p* = 0.012) [75]. The most commonly used ACEi in this meta‐analysis was ramipril, and around two‐thirds of patients were also receiving beta‐blockers (most commonly bisoprolol). Additionally, the prevention of the onset of AF with beta‐blocker plus ACEi combination treatment has also been observed in patients with HF, with a relative risk reduction (RRR) of 27% (95% CI 14–38; *p* < 0.001) [76].

## 6. HF

About 56 million people globally have HF, corresponding to an age‐standardized incidence of 711.9 per 100,000 population [77]. The morbidity associated with HF is substantial, with the age‐standardized years lived with disability (YLDs) of 63.9 per 100,000 population [77]. Efforts are therefore focused not only on the treatment of acute and chronic HF and secondary prevention of complications [78, 79] but also on the primary prevention of HF [80]. Since hypertension is the main risk factor for developing HF [78], controlling BP is essential for the primary prevention of HF (see prior section on hypertension).

Guidelines provide separate recommendations for the management of HFrEF (defined as an LVEF ≤ 40%), HF with mildly reduced ejection fraction (HFmrEF; LVEF 41%–49%), HF with preserved ejection fraction (HFpEF), and acute HF (acute decompensated HF, acute pulmonary oedema, isolated right ventricular failure, and cardiogenic shock) [78]. It is worth noting that hypertensive status (hypertensive vs. normotensive) does not usually alter the approach to the management of a patient with HFrEF and patients receiving optimal medical therapy for HFrEF rarely experience uncontrolled BP [78]. This is attributed to the reduced cardiac output counterbalancing hypertension‐associated systemic vascular resistance [19].

Optimal therapy of HFrEF (New York Heart Association [NYHA] Class II–IV) includes the use of all of the following agents together: an ACEi or angiotensin receptor–neprilysin inhibitor (ARNI) + a beta‐blocker + a mineralocorticoid receptor antagonist (MRA) + a sodium–glucose Cotransporter 2 (SGLT2) inhibitor (e.g., dapagliflozin and empagliflozin) + a loop diuretic (to treat fluid retention) [78]. The effectiveness of ACEis, beta‐blockers, MRAs, and the two SGLT2 inhibitors in reducing the risk of HF hospitalization and death in individuals with HFrEF is very well established [78]. A recent network meta‐analysis confirmed that the most effective combination for reducing all‐cause mortality was an ACEi + beta‐blocker + MRA + SGLT2 inhibitor (risk ratio [RR] 0.46; 95% CI 0.32–0.66), for reducing cardiovascular death was an ACEi + beta‐blocker + MRA + vericiguat (RR 0.34; 95% CI 0.12–0.90), and for reducing HHF was an ACEi + beta‐blocker + MRA + SGLT2 inhibitor and an ACEi + beta‐blocker + MRA + ivabradine (both RR 0.27; 95% CI 0.18–0.39) [81].

Patients with HFmrEF (LVEF 41%–49%) can be treated with either an RAS inhibitor (ACEis, ARBs, or ARNIs), beta‐blocker, or MRA; however, the ESC guidelines consider the strength of evidence for these agents (Class IIb evidence) to be inferior to the evidence for the use of diuretics and SGLT2 inhibitors (Class I evidence) [79]. Patients with HFpEF frequently have comorbid hypertension since hypertension is the main cause/precursor of HFpEF [19, 78].

As recommended by the 2023 ESH and 2024 ESC guidelines, treatment of hypertension with all major antihypertensive drug classes (ACEis or ARBs, BBs, CCBs, and thiazide/thiazide‐like diuretics), together with SGLT2 inhibitors, is recommended in patients with HFpEF [2, 19]. With respect to beta‐blockers, a recent meta‐analysis indicated that while beta‐blockers significantly reduce all‐cause mortality in HFpEF, they have no significant effect on rehospitalization for HF or its composite with all‐cause mortality [82]. A similar conclusion for better mortality outcomes but not lower risk of HF hospitalization was made in a post hoc analysis of data from four RCTs in patients with HFmrEF/HFpEF [83].

As described above, patients with HF receive combination pharmacological therapy, among which ACEi and beta‐blocker combinations are included. Although European guidelines on HF make no specific mention of the use of an SPC with an ACEi plus a beta‐blocker, as stated in the section on hypertension, an SPC is usually preferred when dual therapy is required. In my view, when an ACEi and a beta‐blocker are prescribed as part of an optimal therapy regimen for a patient with HF (with or without hypertension), an SPC may be appropriate.

### 6.1. Beta‐Blocker + ACEi Combination

Beta‐blockers and ACEis are two drug classes that play an essential role in the optimal management of chronic HF, especially HFrEF [89]. No data are available for the specific combination of bisoprolol plus ramipril (plus other standard HF therapies). However, several studies of bisoprolol for HF included a large majority of patients treated with an ACEi, as discussed further below.

### 6.2. Beta‐Blockers

Bisoprolol treatment in patients with HFrEF effectively lowers all‐cause mortality, as seen in the placebo‐controlled CIBIS‐II study [84]. While the first CIBIS trial, which preceded CIBIS‐II, did not show a significant benefit of bisoprolol over placebo [86], a meta‐analysis of CIBIS and CIBIS‐II confirmed that bisoprolol treatment is associated with a significant reduction in mortality [85] (Table 2). In this meta‐analysis, bisoprolol was also associated with significant reductions in cardiovascular death and sudden death, and the composite outcome of hospitalization and death, versus placebo [85]. Importantly, the majority of patients (90%–100%) in these pivotal CIBIS trials received concomitant treatment with an ACEi (and diuretic) [84, 86]. Post hoc analyses of the CIBIS trials identified other characteristics of bisoprolol treatment for HF, including the fact that mortality and hospitalization outcomes are improved in patients who remain on treatment [90], and these outcomes are superior when bisoprolol is given in combination with an ACEi (enalapril) [91] (Table 3).

**Table 2:** Bisoprolol in the management of heart failure with reduced ejection fraction: Pivotal studies.

Study	Study design (mean follow‐up)	HF study entry criteria	Bisoprolol	Control	Background treatment (both arms)	Mortality	Hospitalization	Composite endpoints	Conclusion
CIBIS‐II [84]	R, DB, PBO‐controlled (1.3 years)	NYHA III/IV LVEF ≤ 35%	1.25–10 mg/day (*n* = 1327)	PBO (*n* = 1320)	Diuretics + ACEi	11.8% vs. 17.3% HR 0.66 (95% CI 0.54–0.81; *p* < 0.0001)	33% vs. 39% HR 0.80 (95% CI 0.71–0.91; *p* = 0.0006)	CV hospitalization or CV death: 29% vs. 35% HR 0.79 (95% CI 0.69–0.90; *p* = 0.0004)	Bisoprolol improves survival in pts with stable HFrEF
CIBIS + CIBIS‐II [85]	Meta‐analysis [84, 86]	NYHA III/IV LVEF < 40%	1.25–10 mg/day (*n* = 1647)	PBO (*n* = 1641)	Diuretics + ACEi (90% of pts in CIBIS received ACEi)	All‐cause: 29.3% RRR (95% CI 17%–40%; *p* = 0.00003) CV death: 28% RRR (95% CI 13%–41%; *p* = 0.001) Sudden death: 37% RRR (95% CI 14%–54%; *p* = 0.003)	25.1% RRR (*p* = 0.001)	Hospitalization or death: 18.4% RRR (95% CI 11%–25%; *p* = 0.00001)	Bisoprolol prevents death, CV death, sudden death, and hospitalization
CIBIS‐III [87, 88]	R, OL (1.2 years)	NYHA II/III LVEF ≤ 35% Age ≥ 65 years	Bisoprolol‐first group^a^: 1.25–10 mg/day (*n* = 505)	Enalapril‐first group^b^: Enalapril 2.5–10 mg bid (*n* = 505)	Diuretics (during mono‐ and combination therapy) MRA only (during monotherapy) MRA and/or ARB (during combination therapy)	All‐cause: HR 0.88 (95% CI 0.63–1.22; *p* = 0.44) CV death: HR 0.97 (95% CI 0.67–1.4; *p* = 0.86)	Hospitalization: HR 0.95 (95% CI 0.76–1.19; *p* = 0.66)	All‐cause mortality or hospitalization: HR 0.94 (95% CI 0.77–1.16)	No significant difference between initiating treatment for HF with bisoprolol or enalapril first, before the two as a combination

Abbreviations: ACEi, angiotensin‐converting enzyme inhibitor; ARB, angiotensin receptor blocker; bid, twice daily; CI, confidence interval; CIBIS, Cardiac Insufficiency Bisoprolol Study; CV, cardiovascular; DB, double blind; EF, ejection fraction; HF, heart failure; HFrEF, heart failure with reduced ejection fraction; HR, hazard ratio; LVEF, left ventricular ejection fraction; MRA, mineralocorticoid receptor antagonist; NYHA, New York Heart Association; OL, open‐label; PBO, placebo; pts, patients; R, randomized; RRR, relative risk reduction.

^a^Patients in this group received bisoprolol monotherapy for 6 months; then, enalapril was added for a further 12 months (combination therapy) [87, 88].

^b^Patients in this group received enalapril monotherapy for 6 months; then, bisoprolol was added for a further 12 months (combination therapy) [87, 88].

**Table 3:** Bisoprolol in the management of heart failure with reduced ejection fraction: Post hoc analyses.

Author, year	Data source	Outcomes of post hoc analyses (bisoprolol vs. PBO)	Conclusion(s)
Funck‐Brentano et al., 2001 [90]	CIBIS‐II	Death as first event: 79 vs. 124 pts; RR 0.61 (95% CI 0.46–0.81; *p* = 0.0006) Nonlethal CV hospitalization as first event: 279 vs. 343 pts; RR 0.79 (95% CI 0.67–0.92; *p* = 0.003)	• Bisoprolol therapy should not be discontinued since bisoprolol reduced mortality and CV‐related hospitalizations vs. PBO in pts remaining on treatment
Funck‐Brentano et al., 2011 [91]	CIBIS‐III	Mortality/all‐cause hospitalization multivariable analyses: Monotherapy vs. combination therapy, HR 58.1 (95% CI 37.9–88.9; *p* < 0.0001); ≥ 50% target bisoprolol dose, HR 2.40 (95% CI 1.79–3.21; *p* < 0.0001); ≥ 50% target enalapril dose, HR 2.46 (95% CI 1.85–3.26, *p* < 0.0001)	• Strongest predictor of outcome was study phase; that is, combination beta‐blocker + ACEi was superior to monotherapy for mortality/all‐cause hospitalization • Dose level reached may be influenced by the order in which drug class is introduced for monotherapy (beta‐blocker first vs. ACEi first)
Krum et al., 2011 [92]	CIBIS‐III	Sudden death (end of study): HR 0.84 (95% CI 0.51–0.38; *p* = 0.487) Progressive pump failure death (end of study): HR 2.39 (95% CI 0.99–5.75; *p* = 0.053) All‐cause death/CHF hospitalization (end of study): HR 0.98 (95% CI 0.74–1.29; *p* = 0.89)	• Trend for nonsignificant reduction in risk of sudden death or pump failure death at the end of the study after starting monotherapy with bisoprolol vs. enalapril before combining the two agents in a combination regimen

Abbreviations: ACEi, angiotensin‐converting enzyme inhibitor; CHF, coronary heart failure; CI, confidence interval; CIBIS, Cardiac Insufficiency Bisoprolol Study; CV, cardiovascular; HR, hazard ratio; PBO, placebo; pts, patients; RR, relative risk.

Similarly, mortality benefit has been observed with other beta‐blockers: carvedilol in the COPERNICUS trial [93, 94] and metoprolol in the MERIT‐HF study [95]. Meta‐analyses comparing the relative effects of different beta‐blockers on cardiovascular, hospitalization, and/or mortality outcomes show mixed results regarding the superiority of one beta‐blocker over another, and results seem to depend on the specific HF characteristics of the patient populations included in these meta‐analyses [50, 96].

CIBIS‐III examined whether the particular antihypertensive class received during an initial 6‐month treatment period for HFrEF, either a beta‐blocker (bisoprolol) or an ACEi (enalapril), influenced mortality and hospitalization outcomes in patients who then went on to receive 6–18 months′ combination treatment with bisoprolol plus enalapril [87] (Table 2). Other antihypertensives could be given during the “monotherapy” and combination therapy phases (e.g., diuretics and/or an MRA). CIBIS‐III confirmed the benefit of bisoprolol/enalapril versus either monotherapy, and that starting treatment with one or the other drug class did not influence rates of mortality (primary outcome), cardiovascular death, or hospitalization [87].

### 6.3. ACEis

Overall, meta‐analyses have confirmed the place in therapy of ACEis for HF, particularly HFrEF, and their adverse event profile is well‐described [89, 97, 98]. The first ACEi to show a large reduction in mortality (40% vs. placebo; *p* = 0.002) was enalapril in the landmark CONSENSUS trial in patients with severe congestive HF receiving conventional therapy (other vasodilators) [99, 100], followed by similar positive findings in the SOLVD trial of patients with HFrEF (EF ≤ 35%) [101].

Subsequently, a handful of studies of ramipril ≤ 20 mg/day in small numbers of patients with established congestive HF were published, illustrating that hemodynamic changes were similar to those for captopril (≤ 150 mg/day) and that patients experienced improvements in NYHA functional class and exercise duration (as reviewed previously [37]). The majority of the evidence for the efficacy of ramipril in HF is from trials in patients with AMI complicated by HF—in particular, the Acute Infarction Ramipril Efficacy (AIRE) study [102, 103], its extension study (AIREX) [104], and a subgroup long‐term follow‐up study (AIRE‐S) [105]. The all‐cause mortality rate was significantly lower in ramipril (*n* = 1014) than placebo (*n* = 992) recipients (17% vs. 23%; RRR 27%; 95% CI 11–40; *p* = 0.002), as was the rate of a first event (death, severe/resistant HF, MI, and stroke; RRR 19%; 95% CI 5–31; *p* = 0.008) after a mean follow‐up of 15 months in AIRE [102] and after a mean follow‐up of 59 months in AIREX (mortality RRR 36%; 95% CI 15–52; *p* = 0.002) [104]. Ramipril was associated with a sustained clinical benefit according to an analysis of long‐term follow‐up data (up to 29.6 years) for AIRE participants from the United Kingdom (*n* = 603) [105]. Life expectancy was longer particularly in patients with than without diabetes (32.1 vs. 5.0 months), hypertension (16.6 vs. 8.3 months), angina (16.2 vs. 5.0 months), prior HF (19.5 vs. 4.9 months), and in older (> 65 years) versus younger (≤ 65 years) patients (11.3 vs. 5.7 months) [105].

Additionally, the pivotal HOPE trial assessed whether ramipril could prevent HF in patients at high risk of developing HF (*n* = 9297) [57]. At enrolment, patients aged ≥ 55 years without HF had CAD (~80% of patients) and/or diabetes (~38%), and at least one other cardiovascular risk factor (~47% had hypertension, ~66% elevated total cholesterol, ~18% low high‐density lipoprotein cholesterol, ~21% microalbuminuria, and ~14% were current smokers). Ramipril reduced the risk of cardiovascular‐related death, MI, or stroke (composite outcome) versus placebo (14.0% vs. 17.8%; RR 0.78; 95% CI 0.70–0.86; *p* < 0.01) and the risk of HF (RR 0.77; *p* < 0.001) [57]. Ramipril also reduced the rate of new‐onset HF from 11.5% to 9.0% (*p* < 0.0001), of HF after MI by 13% (RR 0.87; 95% CI 0.66–1.15), and of HF in patients who did not have an MI (RR 0.78; 95% CI 0.62–0.97) [106].

## 7. Synthesis of the Evidence for Guideline and Clinical Practice

The pharmacodynamic, dose–response, and treatment adherence/persistence evidence from landmark clinical trials and meta‐analyses of RCTs discussed in this article supports guideline‐recommended beta‐blocker/ACEi combination therapy for reducing BP levels and mortality in patients with hypertension and those with CAD, AF, or HF [2, 6, 14, 19, 23, 26, 44, 45, 75, 76, 87, 90, 91]. The evidence supports the use of an SPC containing bisoprolol and ramipril for the clinical management of patients with a high or very high CV risk profile, as recommended by the guidelines for hypertension [19], CAD [42], AF [60], and HF [79]. Large RCTs are needed to compare cardiovascular outcomes with beta‐blocker/ACEi SPCs with ACEi/CCB or ACEi/thiazide SPCs. More data are also needed on long‐term metabolic, renal, and arrhythmic effects of beta‐blocker/ACEi SPCs.

## 8. Beta‐Blocker + ACEi SPCs: Summary of Availability and Limitations

Two SPCs containing a beta‐blocker and ACEi (bisoprolol/perindopril and bisoprolol/ramipril) are currently available in Europe [6, 8]; no beta‐blocker/ACEi SPCs have yet received approval from the US Food and Drug Administration. Thus, the product choice of beta‐blocker/ACEi SPCs is very limited. Even where available, SPC uptake in clinical practice remains low relative to guideline recommendations, due to prescribing inertia, lack of reimbursement, and familiarity with free‐form combinations [107–109]. Both currently available SPCs are marketed with multiple dose strengths that cover typical titration steps in hypertension, providing effective therapeutic options for patients not achieving BP targets. However, the fixed‐dose ratio titration limits of a beta‐blocker/ACEi SPC offer less flexibility than the individual components, which can be independently titrated to achieve guideline‐driven target doses in HFrEF [110–112]. Moreover, adverse events induced by individual antihypertensive agents (e.g., hypotension and dizziness with beta‐blockers and hyperkalemia with ACEi) are easier to respond to by adjusting medication doses than by using an SPC. The World Health Organization recommends an SPC as initial treatment for all patients with hypertension requiring pharmacotherapy [11], and international guidelines emphasize that most patients, regardless of age, require combination antihypertensive therapy to achieve BP control [22, 113, 114]. Nevertheless, evidence suggests that patients aged ≥ 65 years with hypertension are treated less aggressively than younger patients [115, 116]. This may be because of evidence suggesting that the benefit of antihypertensive therapy is likely to be modified by age as well as frailty; ACEi has been linked to hypotension and a higher risk of falls in frail older patients [117], while higher doses of beta‐blockers may increase the risk of hip/femur fracture [118], and the benefits of more intensive BP control appear to be mitigated by advanced physical frailty and cognitive decline [119, 120]. Controversially, other research has found no association between antihypertensive treatment with beta‐blockers and ACEi and fracture risk [121, 122]. Additionally, some studies suggest that beta‐blockers and ACEi are associated with a reduced fracture risk [123–125], while intensive BP‐lowering treatment (SBP target < 120 mmHg) did not increase the risk of falls or nonspine fractures in patients aged 40–79 years with Type 2 diabetes compared with those receiving standard BP‐lowering treatment (SBP target < 140 mmHg) in the Action to Control Cardiovascular Risk in Diabetes (ACCORD) randomized trial [126].

## 9. Conclusions

Beta‐blockers and ACEis are antihypertensive agents with clinical benefits beyond BP control, demonstrating therapeutic effectiveness in reducing cardiovascular risk and adverse cardiovascular outcomes while reducing mortality in several prevalent CVDs encountered daily in clinical practice. Therapeutic adherence and treatment regimen simplification are two key aspects to managing patients with hypertension, CAD, AF, and/or HF. The SPC of a beta‐blocker plus an ACEi, such as bisoprolol plus ramipril, may represent an additional option where these two drug classes should be combined for optimal medical therapy.

## Author Contributions

Agostino Virdis conceived and defined the scope of this review and read and critically reviewed the drafts for intellectual content.

## Funding

This work was funded by Sandoz (10.13039/100011218). Open access publishing facilitated by Universita degli Studi di Pisa, as part of the Wiley ‐ CRUI‐CARE agreement.

## Disclosure

The author approved the final draft for submission.

## Conflicts of Interest

The author declares no conflicts of interest.

## Data Availability

Data sharing is not applicable to this article as no datasets were generated or analyzed in the preparation of this manuscript.
